# Comparative Analysis of Diagnostic Performance: Differential Diagnosis Lists by LLaMA3 Versus LLaMA2 for Case Reports

**DOI:** 10.2196/64844

**Published:** 2024-11-19

**Authors:** Takanobu Hirosawa, Yukinori Harada, Kazuki Tokumasu, Tatsuya Shiraishi, Tomoharu Suzuki, Taro Shimizu

**Affiliations:** 1 Department of Diagnostic and Generalist Medicine Dokkyo Medical University Shimotsuga Japan; 2 Department of General Medicine Okayama University Graduate School of Medicine, Dentistry and Pharmaceutical Sciences Okayama Japan; 3 Higashinihonbashinaika Clinic Tokyo Japan; 4 Ubie, Inc Tokyo Japan; 5 Department of Hospital Medicine Urasoe General Hospital Okinawa Japan

**Keywords:** artificial intelligence, clinical decision support system, generative artificial intelligence, large language models, natural language processing, NLP, AI, clinical decision making, decision support, decision making, LLM: diagnostic, case report, diagnosis, generative AI, LLaMA

## Abstract

**Background:**

Generative artificial intelligence (AI), particularly in the form of large language models, has rapidly developed. The LLaMA series are popular and recently updated from LLaMA2 to LLaMA3. However, the impacts of the update on diagnostic performance have not been well documented.

**Objective:**

We conducted a comparative evaluation of the diagnostic performance in differential diagnosis lists generated by LLaMA3 and LLaMA2 for case reports.

**Methods:**

We analyzed case reports published in the *American Journal of Case Reports* from 2022 to 2023. After excluding nondiagnostic and pediatric cases, we input the remaining cases into LLaMA3 and LLaMA2 using the same prompt and the same adjustable parameters. Diagnostic performance was defined by whether the differential diagnosis lists included the final diagnosis. Multiple physicians independently evaluated whether the final diagnosis was included in the top 10 differentials generated by LLaMA3 and LLaMA2.

**Results:**

In our comparative evaluation of the diagnostic performance between LLaMA3 and LLaMA2, we analyzed differential diagnosis lists for 392 case reports. The final diagnosis was included in the top 10 differentials generated by LLaMA3 in 79.6% (312/392) of the cases, compared to 49.7% (195/392) for LLaMA2, indicating a statistically significant improvement (*P*<.001). Additionally, LLaMA3 showed higher performance in including the final diagnosis in the top 5 differentials, observed in 63% (247/392) of cases, compared to LLaMA2’s 38% (149/392, *P*<.001). Furthermore, the top diagnosis was accurately identified by LLaMA3 in 33.9% (133/392) of cases, significantly higher than the 22.7% (89/392) achieved by LLaMA2 (*P*<.001). The analysis across various medical specialties revealed variations in diagnostic performance with LLaMA3 consistently outperforming LLaMA2.

**Conclusions:**

The results reveal that the LLaMA3 model significantly outperforms LLaMA2 per diagnostic performance, with a higher percentage of case reports having the final diagnosis listed within the top 10, top 5, and as the top diagnosis. Overall diagnostic performance improved almost 1.5 times from LLaMA2 to LLaMA3. These findings support the rapid development and continuous refinement of generative AI systems to enhance diagnostic processes in medicine. However, these findings should be carefully interpreted for clinical application, as generative AI, including the LLaMA series, has not been approved for medical applications such as AI-enhanced diagnostics.

## Introduction

### Artificial Intelligence in Medicine

The concept of artificial intelligence (AI) dates back to the 1950s when the potential for machines to mimic human intelligence first began to be explored [1]. Since then, AI technologies, particularly in areas such as neural networks, natural language processing (NLP), and large language models (LLMs), have advanced substantially. These advancements have been driven by significant computational developments and the vast data available in the digital world. Recently, access to these technologies has also become more straightforward, requiring less specific knowledge and fewer resources.

In the realm of AI, neural networks form a foundational concept. These networks mimic the complex interconnections of neurons in the human brain, featuring synapse-like connections that facilitate dynamic learning and adoption. Unlike traditional technologies that rely on static algorithms, neural networks are designed to iteratively adjust the connections between nodes [2]. NLP enables computers to understand and process human language, facilitating tasks such as text translation, voice command response, and data extraction from complex sources. LLMs, advanced forms of NLP, train on extensive corpora of text to generate coherent and contextually relevant text [3]. These technologies have enabled complex models to achieve improved performance and address challenges that traditional approaches cannot handle, such as analyzing large volumes of data to identify patterns that may not be visible to human analysts.

These advancements are now widespread across various sectors, notably in the medical field. Generative AI systems, such as the GPT series developed by OpenAI, Google’s Gemini, and LLaMA, have demonstrated considerable value in research, education, and potential future clinical applications [4,5]. They have the potential to support medical professionals, patients, and their families, by aiding them in making informed clinical decisions based on comprehensive data analysis.

### Generative AI in Medicine

In the medical field, generative AI has been pivotal in advancing diagnostic processes, developing treatment protocols, enabling personalized medicine, and managing patient care [6]. By analyzing vast datasets, generative AI uncovers patterns not immediately obvious to medical professionals, providing crucial insights that lead to improved patient outcomes. For example, generative AI systems are instrumental in enhancing clinical decision-making, optimizing clinical workflows, and improving patient outcomes [7]. Specifically, in diagnosis, generative AI enhances the medical interview process by visualizing the patient’s perspective [8], expands the scope of differential diagnosis lists, and supports clinical reasoning [9,10].

### From LLaMA2 to LLaMA3

The evolution of generative AI systems has been notably rapid, primarily due to their ability to integrate user feedback and continuously update from expanded datasets. This iterative improvement is evident in the progression from GPT-3 to GPT-4, and more recently to GPT-4o and OpenAI o1 [11,12]. Similarly, other systems such as Bard have evolved into more advanced versions such as Gemini and Gemini Advanced [13]. In this dynamic landscape, the LLaMA series has also undergone upgrades, moving from LLaMA2 to LLaMA3, enhancing their capabilities [14].

### Generative AI in Diagnostics

In diagnostics, generative AI systems have the potential to enhance diagnostic performance. These systems excel at processing and interpreting complex clinical data from diverse courses such as electronic health records, imaging studies, and genomic data. Notably, the GPT series has demonstrated considerable diagnostic performance in medical benchmarks and complex case analyses [15]. While significant strides have been made, studies have indicated that other LLM models, such as LLaMA2, require substantial refinement for optimal application in diagnostics [16,17]. Our own study revealed that the diagnostic performance by LLaMA2 was inferior to those of ChatGPT-4 and Gemini for case-report series [18]. This necessitates ongoing development to improve model accuracy and reliability, ensuring they meet clinical standards and effectively support diagnostic decision-making.

### Study Aims

Despite these advancements, the diagnostic capabilities of updated AI models such as LLaMA3 have not been comprehensively explored. There is a particular lack of comparative studies examining the improvements in diagnostic performance from LLaMA2 to LLaMA3. In this context, our study aims to fill this gap by assessing and comparing the diagnostic performance of LLaMA3 to LLaMA2. Specifically, we intend to evaluate their effectiveness in generating differential diagnosis lists for comprehensive case reports. This comparison will explain the evolutionary benefits of the generative AI system upgrade and their practical implications in future diagnostics.

## Methods

### Overview

**Figure 1:**
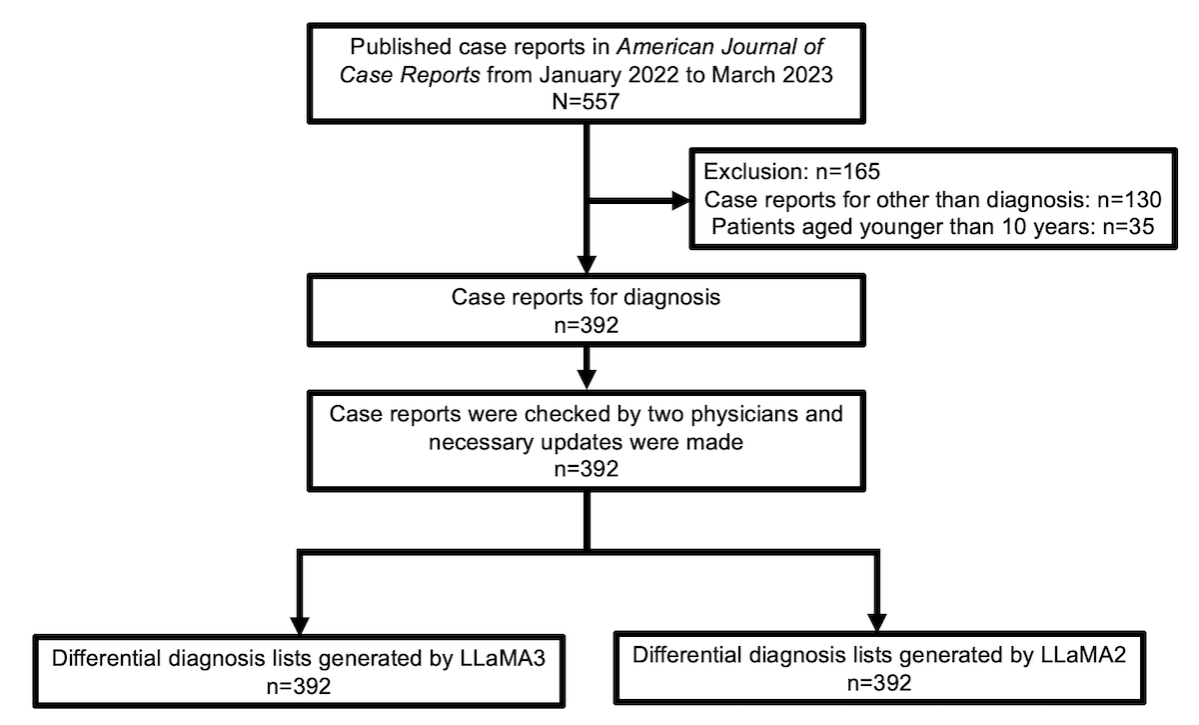
The flowchart, including preparing case reports and generating differentials.

This was an experimental study using publicly available generative AI systems and published case reports. The entire study was conducted at the Department of Diagnostic and Generalist Medicine (General Internal Medicine), Dokkyo Medical University, Japan. This study consisted of four components, including preparing case reports, generating differentials by AIs, evaluating the differentials, and analysis. The flowchart, including preparing case reports and generating differentials, is shown in Figure 1.

### Ethical Considerations

We used published case reports; therefore, ethical approval was inapplicable.

### Case Reports

We used the dataset from our previous research [18]. Our inclusion criteria included case reports published in the *American Journal of Case Reports* from January 2022 to March 2023. We excluded nondiagnostic cases and pediatric cases. These exclusion criteria were adopted from a previous study for a clinical decision support system [19]. For the included case reports, we refined the text data for input. This process involved extracting the clinical narrative from each case report up until the stated final diagnosis. We carefully removed any sections that included clinical assessments or subjective interpretations by the authors to minimize the risk of biasing the AI’s output. This editing was designed to ensure that the input to the AI models was focused on clinical information essential for generating accurate differential diagnoses. The final diagnoses were typically written by the authors. The main investigator, TH, conducted this process, which was validated by another coinvestigator, YH. Details of preparing case reports are shown in Multimedia Appendix 1.

### Differentials Generated by AIs

We used popular generative AI systems developed by Meta AI, LLaMA3 and LLaMA2, to generate differentials. LLaMA3 offers 8B and 70B versions, while LLaMA2 includes 7B, 13B, and 70B versions. For our study, we used the most capable models, the 70B versions. The main investigator, TH, inputted the same cases into both LLaMA3 and LLaMA2 using the same prompt to generate the top 10 differential diagnosis lists.

Both LLaMA3 and LLaMA2 allowed for several adjustable settings to control the output, including temperature, top-P (nucleus), and max tokens. All parameters were set uniformly for this study. The temperature was set at a low value of 0.01 to prioritize predictability in the model’s output. This setting reduces the randomness and creativity of the responses, favoring deterministic and consistent results ideal for medical diagnostics where accuracy is paramount. The top-P parameter was set at 1, allowing for the broadest selection of words while maintaining focus on relevant content, crucial for generating precise differential diagnoses. Lastly, the max tokens were limited to streamline the output, ensuring that the AI focuses on generating concise, relevant differential-diagnosis lists. Table 1 illustrates the key characteristics of the methods to generate differentials, including adjustable parameters and the prompts. The details of methods to generate differentials, including adjustable parameters and system prompts are shown in Multimedia Appendix 2.

**Table 1:** The key characteristics of the methods to generate differentials include adjustable parameters and the prompts in this study.

	LLaMA3	LLaMA2
Developer	Meta AI	Meta AI
Version	70B	70B
Release date	April 2024	July 2023
Access date	May 2024	May 2024
Prompt	“Tell me the top 10 suspected illnesses for the following case: (copy and paste the case)”	“Tell me the top 10 suspected illnesses for the following case: (copy and paste the case)”
Temperature	0.01	0.01
Max tokens	500	500
Top-P	1	1

### Evaluation

Two expert physicians, T Shiraishi and T Suzuki, independently evaluated the differentials. We adopted a binary approach to evaluate whether the final diagnosis was included in the differential diagnosis lists. When the lists included the final diagnosis, their rankings were also evaluated. To ensure consistency and objectivity in evaluations, any discrepancies between the initial assessments by T Shiraishi and T Suzuki were resolved through a consensus meeting involving a third expert physician, KT. To enhance the reliability of our evaluation process, we considered implementing a κ statistic to quantify interevaluator agreement. All evaluators were blinded to which AI system generated the differentials to prevent bias. The details of evaluation methods are shown in Multimedia Appendix 3.

### Analysis

In this study, diagnostic performance was defined as the inclusion of the final diagnosis in the differential diagnosis lists.

### Outcome

We defined the primary outcome as the ratio of cases where the final diagnosis was included in the top 10 differential diagnosis lists generated by LLaMA2 or LLaMA3. The denominator was the total number of cases. The numerator was the number of cases in which the final diagnosis was included in the lists. The secondary outcomes were defined as the ratios of whether the final diagnosis was included in the top 5 differential diagnosis lists and as the top diagnosis, generated by LLaMA2 or LLaMA3. We defined the primary outcome and the secondary outcomes as overall diagnostic performance. Additionally, interrater reliability between the physicians’ evaluation for the differential diagnosis lists was calculated as the Cohen κ coefficient.

### Exploratory Analysis

The dataset for this analysis comprised cases sourced from a broad spectrum of medical specialties. Each case report was tagged with one to six relevant medical specialties, ensuring a comprehensive representation of the diverse areas in medicine. These specialties were included as part of the standardized metadata attached to each case report, facilitating an organized and targeted analysis. In this study, we included only those specialties that were tagged in at least 10 different case reports.

The exploratory analysis involved quantifying the number of cases correctly diagnosed within each specialty and calculating the ratio of cases for each specialty where the final diagnosis was included in the top 10 differential diagnosis lists generated by LLaMA3 or LLaMA2. The denominator was the total number of cases for each specialty. The numerator was the number of cases in which the final diagnosis was included in the lists. Additionally, we calculated 95% CIs for each ratio to assess the precision of our estimates.

### Statistical Analysis

Data were analyzed using R software (version 4.2.2; The R Foundation for Statistical Computing). Descriptive statistics for categorical or binary variables were summarized as numbers and percentages. We compared categorical data using the chi-square test to determine statistical significance. All tests were 2-sided, and a *P* value of <.05 was considered to indicate statistical significance. For assessing interrater reliability, the Cohen κ coefficient was computed using the “irr” package in R. The interpretation of Cohen κ was as follows: a value below 0.4 indicates poor agreement; values between 0.41 and 0.75 suggest fair to good agreement; and values from 0.75 to 1 reflect very good to excellent agreement [20].

## Results

### Overview Cases

A total of 392 cases were included in this study. LLaMA3 and LLaMA2 generated differentials for all cases. The representative differentials are shown in Table 2. The dataset of cases, differentials by LLaMA3 and LLaMA2, and the final diagnoses are shown in Multimedia Appendix 4. The cases encompassed a broad range of medical specialties, with the highest number of cases in general internal medicine and surgery, as detailed in Table 3.

**Table 2:** The representative differentials generated by LLaMA3 and LLaMA2.

Final diagnosis	LLaMA3	LLaMA2
Dementia with Lewy bodies	Lewy body dementia Parkinson disease Dementia with Lewy bodies Alzheimer disease Rapid eye movement sleep behavior disorder Neuroleptic malignant syndrome Serotonin syndrome Delirium Frontotemporal dementia Vascular dementia	Parkinson disease Dementia with Lewy bodies Alzheimer disease Vascular dementia Frontotemporal dementia Huntington disease Creutzfeldt-Jakob disease Wernicke-Korsakoff syndrome Mild cognitive impairment Schizophrenia
Infectious myositis	Necrotizing fasciitis Sepsis Cellulitis Myositis Pyomyositis Abscess Osteomyelitis Endocarditis Bacteremia Gas gangrene	Infectious shock Sepsis Bacterial arthritis Muscle abscess Necrotizing fasciitis Cellulitis Pyomyositis Osteomyelitis Septicemia Multiorgan dysfunction syndrome
Wandering spleen	Wandering spleen Splenoptosis Intestinal malrotation Pancreatic heterotopy Gastric volvulus Midgut volvulus Internal hernia Ovarian torsion Uterine fibroid Pelvic inflammatory disease	Chronic pancreatitis Pancreatic neuroendocrine tumor Splenic tumor Intestinal obstruction Chronic appendicitis Pelvic inflammatory disease Endometriosis Ovarian cyst Gastrointestinal stromal tumor Mesenteric ischemia

**Table 3:** Medical specialties in all cases and those correctly identified solely by LLaMA3 and LLaMA2.

Medical specialty^a^	All cases (N=392), n	Cases correctly identified solely by LLaMA3 (N=312), n (%, 95% CIs)	Cases correctly identified by LLaMA2 (N=195), n (%, 95% CIs); improvement ratios
Surgery	67	50 (74.6, 72.5-77)	34 (50.7, 49-52.5)
General internal medicine	64	55 (85.9, 83.7-88.2)	35 (54.7, 52.9-56.5)
Infectious diseases	55	48 (87.3, 84.8-89.7)	39 (70.9, 68.7-73.1)
Cardiology	49	38 (77.6, 75.1-80)	20 (40.8, 39.1-42.6)
Neurology	42	37 (88.1, 85.3-90.9)	23 (54.7, 52.5-57)
Urology	40	34 (85, 82.1-87.9)	23 (57.5, 55.2-59.8)
Oncology	32	26 (81.3, 78.1-84.4)	19 (59.4, 56.7-62)
Metabolic diseases	32	26 (81.3, 78.1-84.4)	19 (59.4, 56.7-62)
Radiology	29	20 (69, 65.9-72)	16 (55.2, 52.5-57.9)
Critical care medicine	27	22 (81.5, 78.1-84.9)	9 (33.3, 31.2-35.5)
Gastrointestinal	27	21 (77.8, 74.5-81.1)	10 (37, 34.7-39.3)
Hematology	22	17 (77.3, 73.6-80.9)	10 (45.5, 42.6-48.3)
Rheumatology	19	14 (73.7, 69.8-77.5)	12 (63.2, 59.6-66.7)
Nephrology	18	14 (77.8, 73.7-81.9)	8 (44.4, 41.4-47.5)
Respiratory	18	15 (83.3, 79.1-87.6)	11 (61.1, 57.5-64.7)
Obstetrics and gynecology	17	11 (64.7, 60.9-68.5)	8 (47.1, 43.8-50.3)
Endocrinology	16	14 (87.5, 82.9-92.1)	7 (43.8, 40.5-47)
Otolaryngology	13	10 (76.9, 72.2-81.7)	4 (30.8, 27.8-33.8)
Orthopedics	10	6 (60, 55.2-64.8)	4 (40, 36.1-43.9)

^a^Each case report was tagged with one to six relevant medical specialties.

### Overall Diagnostic Performance

The final diagnosis was included in the top 10 differentials generated by LLaMA3 in 79.6% (312/392) of the cases, compared to 49.7% (195/392) for LLaMA2, indicating a statistically significant improvement (*P*<.001). Additionally, LLaMA3 showed higher performance in including the final diagnosis in the top 5 differentials, observed in 63% (247/392) of cases, compared to LLaMA2’s 38% (149/392, *P*<.001). Moreover, the final diagnosis was accurately identified as the top diagnosis by LLaMA3 in 33.9% (133/392) of cases, significantly higher than the 22.7% (89/392) achieved by LLaMA2 (*P*<.001). The overall diagnostic performance of LLaMA3 and LLaMA2 is shown in Table 4. We observed fair to good agreement between physicians’ evaluations for the differential diagnosis lists, with a κ coefficient of 0.69, indicating concordance in 84.2% (660/784) of cases.

**Table 4:** Overall diagnostic performance of LLaMA3 and LLaMA2.

Diagnostic performance	LLaMA3, n/N (%)	LLaMA2, n/N (%)	*P* value^a^
The ratio of whether the final diagnosis was included in the top 10 differential diagnosis lists	312/392 (79.6)	195/392 (49.7)	<.001
The ratio of whether the final diagnosis was included in the top 5 differential diagnosis lists	247/392 (63)	149/392 (38)	<.001
The ratio of whether the final diagnosis was included as the top diagnosis	133/392 (33.9)	89/392 (22.7)	<.001

^a^*P* value from chi-squared test.

### Exploratory Analysis by Medical Specialty

**Figure 2:**
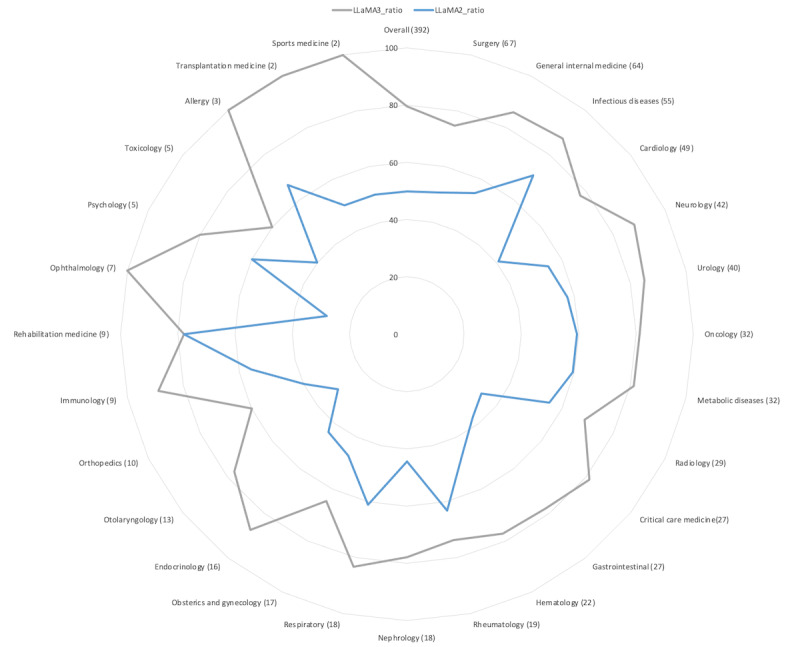
Radar chart illustrating the improvement ratios for the inclusion of the final diagnosis within the top 10 differential diagnosis lists generated by LLaMA2 and LLaMA3, across various medical specialties. Each axis on the radar chart represents a specific medical specialty. The numerical values adjacent to each specialty name reflect the total number of cases analyzed within that specialty, providing context for the observed performance metrics.

The exploratory analysis across various medical specialties revealed variations in diagnostic performance with LLaMA3 consistently outperforming LLaMA2 in almost all fields. All specialties showed improvements of more than 10% from LLaMA2 to LLaMA3, with nonoverlapping 95% CIs, indicating statistically significant enhancements. Specifically, critical care medicine, gastrointestinal, endocrinology, and otolaryngology exhibited remarkable improvements of more than 40% from LLaMA2. Conversely, infectious diseases, radiology, and obstetrics and gynecology showed the least improvements, with about a 10% increase from LLaMA2 to LLaMA3. Other specialties exhibited moderate improvements with 20%-30%. Ophthalmology demonstrated the highest accuracy with 71.4% (5/7) of cases correctly identified, followed by otolaryngology at 61.5% (8/31). Lower accuracy was observed in specialties such as rehabilitation medicine at 11.1% (1/9) and rheumatology at 15.8% (3/19). Other specialties such as general internal medicine and surgery showed moderate performance with accuracies of 34.4% (22/64) and 28.4% (19/67), respectively. Table 3 details the breakdown of medical specialties, showing the total number of cases and those correctly identified by LLaMA3 and LLaMA2 in all cases and those correctly identified solely by LLaMA3. Figure 2 presents a radar chart illustrating the ratio of cases for each specialty where the final diagnosis was included in the top 10 differential diagnosis lists generated by both LLaMA3 or LLaMA2.

## Discussion

### Principal Results

This study demonstrated that the LLaMA3 model significantly outperforms LLaMA2 in overall diagnostic performance, showing almost 1.5-fold improvement. Specifically, the inclusion rate of the final diagnosis in the top 10 differentials rose from 50% to 80%. This substantial enhancement reflects marked advancements within the LLaMA series over a relatively short period.

These enhancements likely come from the implementation of more advanced algorithms and more robust training datasets, highlighting the rapid evolution of generative AI capabilities in medical diagnostics. The significantly higher inclusion rates of the final diagnosis in the top 10, top 5 differentials, and the top diagnosis by LLaMA3 indicate that its model has been finely tuned for greater precision in analyzing complex medical cases. This tuning suggests that LLaMA3 is more adept at incorporating clinical nuances and recognizing a diverse range of symptoms, which is critical for generating accurate differential diagnoses in real-world clinical settings.

### Model Bias and Generalizability

While this study leverages data from a single journal, it is crucial to consider how this might limit the generalizability of the findings. The cases predominantly represent complex or rare medical scenarios, which might not fully represent routine clinical situations found across diverse health care systems [21]. This focus could skew the AI’s performance, suggesting that while LLaMA3 shows promise, its effectiveness in general practice remains to be validated in a more varied clinical context.

### Practical Implementation and Challenges

Integrating LLaMA3 into clinical practice presents several challenges that require careful consideration. The foremost is regulatory approval, as generative AI, including the LLaMA series, has not yet been approved for direct clinical applications such as AI-enhanced diagnostics. Regulatory hurdles can significantly delay or impede the practical application of innovative technologies. Furthermore, clinician trust in AI decision-making is vital and requires the AI to be not only effective but also transparent in how decisions are derived. Clinicians must be able to comprehend how decisions are derived to confidently integrate AI recommendations into their workflow.

The computational demands of running sophisticated models such as LLaMA3 also pose a significant challenge. High-performance computing resources, such as Graphics Processing Units or cloud-based solutions, are essential to operate these advanced AI systems effectively, which could limit their deployment in resource-constrained settings.

### Future Research and Development

To facilitate the effective integration of AI such as LLaMA3 into health care workflows, ongoing training with real-world data and continuous feedback from clinical use are indispensable. This iterative process will help ensure that the AI remains accurate and adapts to evolving medical standards. Exploring multimodal AI that incorporates text and image data from electronic health records could enhance diagnostic accuracy. Future studies should focus on integrating these systems with routine health care workflows to assess their practical utility and acceptance among health care providers. Additionally, addressing potential biases in AI decision-making and ensuring adherence to ethical health care standards are crucial for gaining acceptance and trust in clinical environments.

### Results From Exploratory Analysis

The exploratory analysis across different medical specialties provided a view of LLaMA3’s performance, which varied across fields. For instance, specialties, including critical care medicine, showed exceptionally high improvements in diagnostic accuracy with LLaMA3. This finding highlights its effectiveness in processing complex clinical courses.

However, the analysis also uncovered areas with modest improvements. For instance, radiology showed small improvements, with about a 10% increase from LLaMA2. This result suggests a need for multimodal AI that can process image data in addition to text data [22]. Multimodal AI enables the simultaneous processing and understanding of multiple forms, including text and image data, which is particularly pertinent for enhancing diagnostic accuracy in radiology.

The variability in these improvements highlights the importance of targeted algorithmic training tailored to the specific demands of each medical specialty. Specialized training datasets that encompass the wide range of scenarios encountered in particular fields could be crucial in enhancing the generative AI’s learning curve and improving its utility in clinical practice. The performance of LLaMA3 varies across medical specialties, with notably high improvement ratios in ophthalmology and otolaryngology, likely due to the distinct and well-defined symptoms associated with conditions treated within these fields. Conversely, specialties such as rehabilitation medicine and rheumatology showed lower improvement ratios, attributed to the complexity of the clinical course and immune responses, posing challenges for the current model’s diagnostic algorithms. A significant factor contributing to the variation in performance is the relatively small number of cases available for some specialties.

### Strengths

A major strength of this study is the controlled comparison of diagnostic performances using identical cases and standardized parameters, providing a clear assessment of improvements from LLaMA2 to LLaMA3. Additionally, the longitudinal assessment of the LLaMA series offers valuable insights into the developmental course of AI models in medical diagnostics. This is particularly notable when contrasted with findings from other AI systems where no improvement was noted over time [23].

### Limitations

#### Overview

There were several limitations concerning study design and generative AI.

#### Limitations for Study Design

First, case reports may not fully reflect real-world clinical cases. This limitation arises because case reports often focus on new or rare diseases, which might not be commonly encountered in typical clinical settings [21]. Second, relying solely on a single case report journal may introduce selection bias. Third, there was no well-established standard to evaluate the diagnostic performance of clinical decision support systems, including the number of differentials and the evaluation methods. For example, a study adopted 5 differentials while another adopted 40 differentials [24,25]. Regarding evaluation methods, some studies used scale-based assessments, while others used binary methods. Qualitative evaluations of the differential diagnosis lists should also be explored in future studies to assess their overall clinical relevance beyond whether the correct diagnosis was included. These variations in evaluation methods were partly due to the complexity of the diagnostic process in real clinical situations [26]. Fourth, we excluded specialties tagged in fewer than 10 different case reports. Therefore, there was a possibility to overlook minor specialties where LLaMA3 did not outperform LLaMA2. Fifth, the variability in sample sizes across specialties in our exploratory analysis might affect the robustness of the conclusions drawn. Additionally, the sensitivity of AI models such as the LLaMA series to variations in input prompts—prompt engineering—is a critical area. There is a potential that even minor prompt changes presented to the AI can significantly influence its diagnostic suggestions, emphasizing the need for standardized prompt protocols to ensure consistent AI performance.

#### Limitations for Generative AI

Generative AI, including the LLaMA series, has not been approved for medical applications such as AI-enhanced diagnostics. Additionally, the optimal prompts and adjustable parameters for medical diagnostics remain unknown. For example, another study used different settings with a temperature of 0.6, top-P of 0.9, and max tokens of 2048 [16], in contrast to our study which used a temperature of 0.01, top-P of 1, and max tokens of 500. Similarly, another study used multiple prompting scenarios, such as chain of thought, few shots, and retrieval augmentations [27], compared to our study with a simple prompt. This difference in prompting complexity could impact the generative AI’s performance. Furthermore, we did not recruit all available generative AI, including the ChatGPT series, Gemini, and Claude 3. Moreover, a critical limitation identified in our study involves the potential for data leakage, where LLaMA3 and LLaMA2 might have been previously exposed to the case reports used in our analysis, thereby influencing their performance artificially. The inherent risk of data leakage cannot be entirely ruled out due to the models’ continuous learning capabilities and the complex nature of their training environments. To mitigate such risks in future studies, we plan to implement rigorous partitioning of data to ensure that no overlap occurs between training and testing datasets. Regarding transparency, although the LLaMA series is often referred to as open-source LLMs, there is an ongoing debate about the openness of generative AIs [28,29]. Finally, the rapid pace of development in generative AI systems suggested that our findings may quickly become outdated as next-generation LLMs emerge.

These limitations could affect generalizability.

### Comparison With Prior Work

#### Comparison With LLaMA2

Following the limitations outlined, our comparative analysis with prior iterations of LLaMA2 highlights the dynamic nature of AI development and its implications on diagnostic accuracy. In our study, the inclusion of the final diagnosis in the top 10 differentials for 49.7% (195/392) of cases represents a decrease from the 54.6% (214/392) observed in our prior study [18]. This variation in performance, a 1%-5% difference, is directly attributable to the adjustments in operational parameters such as temperature, max tokens, and top-P. These findings highlight how seemingly minor tweaks in AI configurations can lead to significant changes in outcome, emphasizing the necessity for continuous optimization based on evolving clinical needs.

Our results not only reflect the critical impact of parameter adjustments on the efficacy and reliability of AI diagnostic outputs but also the importance of tailoring these settings to specific diagnostic tasks within clinical environments. The ongoing research and development efforts are vital as they contribute to refining these parameters to enhance the performance of AI systems in real-world settings. Table 5 details the diagnostic performance and key characteristics of LLaMA2 compared to the previous study, illustrating these points and showing the progression within the LLaMA series.

**Table 5:** Diagnostic performance and key characteristics of LLaMA2 compared to a previous study.

	LLaMA2 in this study	LLaMA2 in the previous study
The ratio of whether the final diagnosis was included in the top 10 differential diagnosis lists, n/N (%)	195/392 (49.7)	214/392 (54.6)
The ratio of whether the final diagnosis was included in the top 5 differential diagnosis lists, n/N (%)	149/392 (38)	177/392 (45.2)
The ratio of whether the final diagnosis was included as the top diagnosis, n/N (%)	89/392 (22.7)	90/392 (23)
Developer	Meta AI	Meta AI
Version	70B	70B
Release date	July 2023	July 2023
Access date	May 2024	August 2023
Prompt	“Tell me the top 10 suspected illnesses for the following case: (copy and paste the case)”	“Tell me the top 10 suspected illnesses for the following case: (copy and paste the case)”
Temperature	0.01	2.49
Max tokens	500	2048
Top-P	1	0.5

#### Comparison With Other Generative AI

From another study involving ChatGPT-3.5, ChatGPT-4, and LLaMA2, inferior performances of LLaMA2 compared to ChatGPT-3.5 and ChatGPT-4 were observed [16]. From the current findings, there is a possibility that results may change due to longitudinal improvements from LLaMA2 to LLaMA3.

Our comparative analysis extends beyond LLaMA2 and LLaMA3 to include contemporary models such as ChatGPT-4 and Gemini, providing a broader perspective on generative AI capabilities. While LLaMA3 has shown notable improvements and closely matches the performance of ChatGPT-4 with a diagnostic accuracy of 86.7% (340/392) in the top 10 differentials [18], it is essential to consider the development timelines and the operational models of these AI systems. Unlike LLaMA3, ChatGPT-4(o) and Gemini Advanced are fee-based models that might have different optimization and deployment strategies, potentially affecting their performance in clinical settings. Moreover, the introduction of newer models such as ChatGPT-4o and OpenAI o1 represents continuous advancements within the generative AI landscape, highlighting the dynamic nature of AI development.

#### Comparison With Other Clinical Decision Support Systems

Expanding on our comparative analysis, we also evaluate LLaMA3 in the context of established clinical decision support systems such as Isabel Pro (developed by Isabel Healthcare). While Isabel Pro has demonstrated a diagnostic retrieval accuracy of 65% for its top 10 differentials, increasing to 87% for the top 40 [25], these figures provide a benchmark for evaluating LLaMA3’s capabilities. Our study’s performance metrics are closely aligned with these established systems, suggesting that LLaMA3 could offer comparable benefits in clinical decision-making. It is crucial to understand the methodologies and metrics used across different systems to ensure a fair and meaningful comparison.

### Conclusions

The results demonstrate that the LLaMA3 model significantly outperforms LLaMA2 per diagnostic performance, with a higher percentage of case reports having the final diagnosis listed within the top 10, top 5, and as the top diagnosis. Overall diagnostic performance improved almost 1.5 times from LLaMA2 to LLaMA3. These findings support the rapid development and continuous refinement of generative AI systems to enhance diagnostic processes in medicine. However, these findings should be carefully interpreted for clinical application, as generative AI, including the LLaMA series, has not been approved for medical applications such as AI-enhanced diagnostics.

## Appendix

Multimedia Appendix 1 The details of preparing case reports.

Multimedia Appendix 2 The details of methods to generate differentials, including adjustable parameters and system prompt.

Multimedia Appendix 3 The details of evaluation methods.

Multimedia Appendix 4 The dataset of cases, differentials, and the final diagnoses, used in our study.

## Abbreviations

AI: artificial intelligence
LLM: large language model
NLP: natural language processing
