# Supplementary material for: Comparative Analysis of Diagnostic Performance: Differential Diagnosis Lists by LLaMA3 Versus LLaMA2 for Case Reports
Source: JMIR Form Res. 2024 Nov 19;8:e64844. doi: 10.2196/64844 (PMC11615545; doi:10.2196/64844)
Supplement: Multimedia Appendix 1 [file formative_v8i1e64844_app1.docx]

To establish a rigorous framework for the selection of case reports, specific inclusion and exclusion criteria were set, aiming to enhance the generalizability of the study outcomes. The cases were meticulously sourced from the *American Journal of Case Reports*, a peer-reviewed journal known for its comprehensive and often complex case reports across various medical disciplines. The structured format of these reports simplifies the extraction of detailed case descriptions, which invariably include the patients' final diagnoses, thus aligning well with the study's objectives.

**Case Selection Process**

A systematic literature search was conducted using PubMed to compile the case reports, employing specific search terms and filters. The search query was structured to include case reports published between January 1, 2022, and March 1, 2023, using the filter "(2022/1/1:2023/3/1[dp]) AND (American Journal of Case Reports[journal])". Here, "[dp]" specifies the publication date range, while "[journal]" identifies the source of the publications.

**Inclusion and Exclusion Criteria**

Inclusion criteria were confined to reports within the specified dates to ensure the most recent clinical findings were considered. The exclusion criteria were applied to omit reports predominantly centered on management strategies lacking in-depth diagnostic details and those involving patients under the age of 10 years. This focus helped streamline the study towards case reports that provided rich diagnostic information suitable for analysis.

**Screening and Selection**

An initial tally identified 557 consecutive case reports. These were subjected to a thorough eligibility review by the primary investigator to ensure adherence to the predefined criteria. The process was audited by another investigator to maintain accuracy and integrity in the study's foundational data. Cases primarily focused on management issues led to the exclusion of 130 reports, while another 35 were omitted due to patient age restrictions. In instances of reports documenting multiple cases, only the first case presented was considered. This screening process resulted in 392 case reports being included for final analysis.

**Data Extraction and Editing**

For the purpose of analysis, each report was edited to succinctly highlight the inputted data and the final diagnoses. Typically, inputted data were extracted from sections of the reports detailing patient history, physical examinations, investigative results, and management approaches leading up to the final diagnosis. Extraneous sections such as titles, introductions, discussions, conclusions, and any assessments not directly relevant to the core case description, along with accompanying tables or figures, were systematically excluded. The final diagnoses were typically defined by the authors. Any discrepancies in the selection or extraction processes were collaboratively discussed between the investigators, TH and YH, until a consensus was achieved, ensuring a high standard of data integrity and fidelity in the study's methodology.
