# Supplementary material for: Comparative Analysis of Diagnostic Performance: Differential Diagnosis Lists by LLaMA3 Versus LLaMA2 for Case Reports
Source: JMIR Form Res. 2024 Nov 19;8:e64844. doi: 10.2196/64844 (PMC11615545; doi:10.2196/64844)
Supplement: Multimedia Appendix 2 [file formative_v8i1e64844_app2.docx]

|  | LLaMA3 | LLaMA2 |
| --- | --- | --- |
|  |  |  |
| **Developer** |  |  |
|  | Meta AI | Meta AI |
| **Available Versions** |  |  |
|  | 8B, 70B | 7B, 13B, 70B |
| **Version in This Study** |  |  |
|  | 70B | 70B |
| **Release Date** |  |  |
|  | April 2024 | July 2023 |
| **Access Date** |  |  |
|  | May 2024 | May 2024 |
| **Prompt** |  |  |
|  | "Tell me the top 10 suspected illnesses for the following case: (copy and paste the case)" | "Tell me the top 10 suspected illnesses for the following case: (copy and paste the case)" |
| **Temperature** |  |  |
|  | 0.01 | 0.01 |
| **Max Tokens** |  |  |
|  | 500 | 500 |
| **Min Tokens** |  |  |
|  | -1 | -1 |
| **Top P** |  |  |
|  | 1 | 1 |
| **Top K** |  |  |
|  | 50 | 50 |
| **Presence Penalty** |  |  |
|  | 0 | 0 |
| **Frequency Penalty** |  |  |
|  | 0 | 0 |
| **System Prompt** |  |  |
|  | You are a helpful assistant. | You are a helpful assistant. |

LLaMA: large language model by Meta AI
