# Supplementary material for: Comparative Analysis of Diagnostic Performance: Differential Diagnosis Lists by LLaMA3 Versus LLaMA2 for Case Reports
Source: JMIR Form Res. 2024 Nov 19;8:e64844. doi: 10.2196/64844 (PMC11615545; doi:10.2196/64844)
Supplement: Multimedia Appendix 3 [file formative_v8i1e64844_app3.docx]

This appendix provides a detailed description of the evaluation methods used to assess the accuracy and clinical relevance of the differential diagnosis lists generated by LLaMA3 and LLaMA2. To assess the accuracy and clinical relevance of the differential diagnosis lists generated by LLaMA3 and LLaMA2, a systematic evaluation process was implemented. Two general internal medicine (GIM) expert physicians, Tatsuya Shiraishi and Tomoharu Suzuki who were not privy to the AI model that produced the lists, conducted the evaluations independently.

**Coding and Review Process**

Each differential diagnosis list was scrutinized against the corresponding final diagnosis documented in the case reports. The physicians employed a binary coding system to classify the relevance and accuracy of each item in the list. A code of "1" was assigned to items that either directly matched the final diagnosis with an acceptable level of specificity or were closely related enough to the final diagnosis that initiating treatment based on these suggestions would not compromise patient safety. Conversely, a code of "0" was given to items that diverged significantly from the final diagnosis, indicating that any treatment derived from these suggestions could be ineffective or potentially harmful. This binary evaluation method was adopted from a previous study for diagnostic accuracy (Krupat E, Wormwood J, Schwartzstein RM, Richards JB. Avoiding premature closure and reaching diagnostic accuracy: some key predictive factors. Medical education. 2017;51(11):1127-37).

**Resolution of Discrepancies**

In instances where the initial evaluations differed between the two reviewing physicians, discrepancies were addressed by consulting a third GIM expert physician, Kazuki Tokumasu. This step ensured an additional layer of scrutiny and consensus, maintaining the integrity and accuracy of the evaluation process.

**Blind Review Protocol**

Anonymity regarding which AI model produced the lists was strictly maintained for the evaluators. This blind review protocol was designed to eliminate any potential bias that could influence the evaluators' judgments based on their knowledge of the model’s capabilities or limitations.
